# Association between statin use and physical performance in home-dwelling older patients receiving polypharmacy: cross-sectional study

**DOI:** 10.1186/s12877-022-02942-7

**Published:** 2022-03-23

**Authors:** Sigbjørn Veddeng, Håkon Madland, Espen Molden, Torgeir Bruun Wyller, Rita Romskaug

**Affiliations:** 1 Faculty of Medicine, University of Oslo, Oslo, Norway; 2 Department of Pharmacy, University of Oslo, Oslo, Norway; 3 Center for Psychopharmacology, Diakonhjemmet Hospital, Oslo, Norway; 4 Institute of Clinical Medicine, University of Oslo, Oslo, Norway; 5 Department of Geriatric Medicine, Oslo University Hospital, Box 4956 Nydalen, NO-0424 Oslo, Norway

**Keywords:** Older adults, Statins, Lipid lowering drugs, Myopathy, drug side effects, Drug-drug interactions, Polypharmacy, Pharmacogenetics, Gait speed, Grip strength

## Abstract

**Background:**

In older patients with polypharmacy and multiple comorbidities, even low grades of statin-associated muscle symptoms may have clinical implications. The aim of this study was therefore to investigate the potential associations between statin use and measures of physical performance and muscle function.

**Methods:**

Participants were aged 70+, treated with at least seven regular systemic medications, and not expected to die or become institutionalized within 6 months. Physical performance measured as gait speed and Short Physical Performance Battery (SPPB) score, and muscle function measured as grip strength, were compared between users and non-users of statins. In the subgroup of statin users, the dose-response relationship was assessed using harmonized simvastatin equivalents adjusted for statin potency, pharmacokinetic interactions and SLCO1B1 c.521 T > C genotype. Multiple linear regression analyses were applied to investigate potential associations between stain use and exposure as independent variables, and physical performance and muscle function as outcomes, adjusted for age, gender, body mass, comorbidity, disability and dementia.

**Results:**

174 patients (87 users and 87 non-users of statins) with a mean (SD) age of 83.3 (7.3) years were included. In analyses adjusted only for gender, grip strength was significantly higher in users than in non-users of statins [regression coefficient (B) 2.7, 95% confidence interval (CI) 1.0 to 4.4]. When adjusted for confounders, the association was no longer statistically significant (B 1.1, 95% CI − 0.5 to 2.7). SPPB and gait speed was also better in statin users than in non-users, but the differences were not statistically significant. In dose-response analyses adjusted for confounders, we found a statistically significant increase in SPPB score (B 0.01, 95% CI 0.00 to 0.02) and gait speed (B 0.001, 95% CI 0.000 to 0.002) per mg increase in simvastatin equivalents.

**Conclusions:**

In contrast to our hypothesis, statin use and exposure was associated with better measures of physical performance and muscle function in older patients with complex drug treatment. The unexpected findings of this cross-sectional, observational study should be further investigated by comparing physical performance before and after statin initiation or statin withdrawal in prospective studies.

**Trial registration:**

ClinicalTrials.gov identifier: NCT02379455, registered March 5, 2015.

## Background

Lipid-lowering drugs are widely used in the treatment and prevention of atherosclerotic disease [1]. The most used lipid-lowering drugs are statins, or 3-hydroxy-3-methylglutaryl-coenzyme A inhibitors. They are generally regarded as safe to use [2], and their benefits are well documented [1]. However, muscular adverse effects of statins are quite common, ranging from myalgia without biochemical changes to myositis or rhabdomyolysis [3]. A systematic review of statin induced myopathy stated that 0.01% of patients taking statins developed rhabdomyolysis, while 10-15% developed myalgia [4]. Statins may be considered appropriate as well as inappropriate in older adults exposed to polypharmacy, depending on the clinical context. Statin use may be appropriate in those who have high cardiovascular risk and a significant expected length of survival, but inappropriate in advanced frailty with physical disability and short expected length of survival. Many explicit prescription tools such as STOPP, TIME and CRIME refer to statins as inappropriate for patients with life expectancy < 2 years or advanced dementia [5].

Although adverse effects like mild myalgia are not generally associated with a measurable decline in muscle strength in healthy individuals [6]**,** potentially serious consequences may occur in older adults who live with frailty. Physiologic ageing processes lead to pharmacokinetic and pharmacodynamic changes and reduced ability to maintain physiologic homeostasis [7]**,** and thus make older adults more vulnerable for adverse drug reactions [8]. For older patients with reduced muscle function and balance, even low grades of statin-associated muscle symptoms may have clinical implications, i.e. a decline in physical function or increased risk of falling.

The risk of statin-induced myopathy is dependent on the plasma concentration (systemic exposure) [9]. Therefore, pharmacokinetic interactions as well as pharmacogenetic variations elevating statin exposure increase the risk of muscular adverse effects. In recent years, a specific influx transporter, OATP1B1, has been of particular interest regarding this topic [10]. OATP1B1, an anion-transporting polypeptide, is located in the sinusoidal membrane of hepatocytes and facilitates the uptake of several drugs, including statins, into the liver [11]. OATP1B1 expression and function is determined by pharmacogenetic variability in *SLCO1B1*, where the *521 T > C* polymorphism is the most frequent variant associated with reduced OATP1B1-mediated uptake of statins from blood to liver [11]. Carriers of the *SLCO1B1 C* variant obtain higher statin exposure and are at increased risk of muscular side effects [10].

Considering that frail older people are more prone to adverse side effects and also more affected by them [12, 13], we need more knowledge of potential risks of impaired muscular function, gait and balance associated with statin use and exposure in this group. Therefore, the aim of this observational study was to investigate possible associations between statin exposure and physical function measured through gait speed and general mobility, and muscle function, measured as grip strength, in frail older adults receiving polypharmacy, adjusting for possible confounding factors. We hypothesized that measures of muscle function and physical performance would be impaired in statin users compared to non-users, and that there would be a dose-response relationship indicating decreasing muscle function and physical performance with increasing statin exposure.

## Material and methods

### Participants

This observational study utilised baseline data from the COOP (Cooperation between geriatricians and general practitioners for improved pharmacotherapy in home-dwelling elderly people receiving polypharmacy) study cohort [14]. The COOP study was a cluster randomised study of collaborative drug reviews in patients 70 years and older who used at least seven different medications and had their medications administered by the home nursing service. Patients were not eligible if they were expected to die or become permanently institutionalised within 6 months or if they were considered as unfit for the study by the family physician. The COOP study population comprised 174 home-dwelling older adults aged 70-102 years. Half the population (*n* = 87) happened to use a statin whereas the remaining 87 did not.

As part of the COOP study [15], patients were given comprehensive information about the study and were included based upon informed consent. All procedures performed in studies involving human participants were in accordance with the ethical standards of Oslo University Hospital and the regional research committee (Regional Committee for Medical and Health Research Ethics South East; reference number 2014/1488) and with the 1964 Helsinki declaration and its later amendments or comparable ethical standards. The study was conducted in accordance with the Basic & Clinical Pharmacology & Toxicology policy for experimental and clinical studies [16].

### Dependent variables

We measured grip strength as indicator of muscle function and gait speed and Short Physical Performance Battery (SPPB) score as indicators of physical performance. Grip strength was measured using hand dynamometry, with three attempts on each hand. The patients were sitting up to the back of a chair, with approximately 90^o^ angle in knees and elbow, the upper arm close to the side of the chest and neutral position in the wrist. No support of the hand or arm was allowed. The highest score of a total of six attempts was used, results measured in kilograms. Gait speed was determined by measuring the normal gait speed in meters per second, the patient walking a distance of 4 m from a static start and with the possibility to walk further 1-2 m after fulfilling the distance. SPPB is a screening tool for physical function in older adults, and combines the results of gait speed, chair stand and balance tests [17]. SPPB score ranges from 0 to 12, where 12 is best. We also dichotomized the physical performance measures according to the most recent European Working Group on Sarcopenia in Older People (EWGSOP) recommendations [18]. According to these recommendations, a SPPB score ≤ 8, gait speed ≤0.8 m/s, or grip strength < 27 kg for men or < 16 kg for women, are indicative of sarcopenia.

### Adjustment variables

We used the Cumulative Illness Rating Scale (CIRS) [19] to quantify the degree of comorbidity. CIRS ranges from 0 to 56, where an increasing score indicates higher comorbidity. To assess severity of dementia we included the Clinical Dementia Rating scale (CDR) [20]. CDR was scored using the sum of boxes method, with scores ranging from 0 to 18, increasing score indicating more severe dementia. The Functional Independence Measure (FIM) [21] was used to grade the degree of disability. FIM ranges from 18 to 126, increasing score indicating higher grade of independence. Body Mass Index (BMI) was obtained from weighing and self-reported height.

### Statin exposure

For the dose-response analyses, we generated a statin exposure variable, taking into account the different statins and their relative potency as well as SLCO1B1 genotype and pharmacokinetic interactions with co-administered drugs known to affect statin concentrations to a clinically relevant extent. When generating the predicted statin exposure variable, equipotent doses was converted using simvastatin as reference, e.g. 10 mg atorvastatin was defined an exposure similar to 20 mg simvastatin, 10 mg rosuvastatin as an exposure similar to 40 mg simvastatin, and 10 mg pravastatin as an exposure similar to 5 mg simvastatin [22]. As pharmacokinetic differences may alter the effective dose of statins, the calculated dose equivalents were corrected for the expected exposure changes of drug-drug interactions. To identify potential pharmacokinetic drug interactions with statins, we searched a database established by the Norwegian Medicines Agency (www.legemiddelinteraksjoner.no) [23]. Three identified interactions were considered of clinical relevance among the included simvastatin-treated patients, and those were amlodipine, diltiazem and amiodarone, which are CYP3A4 inhibitors increasing serum levels of simvastatin about 1.5-fold [24–26], 3-fold [27, 28], and 1.7-fold [29], respectively. For atorvastatin, pravastatin and rosuvastatin, no clinically relevant interactions were identified in the database searches.

### SLCO1B1 genotyping

*SLCO1B1* is the gene encoding OATP1B1, in which a specific single nucleotide polymorphism (SNP), the *c.521 T > C* variant (rs4149056), decreases the efficiency of OATP1B1-mediated influx [30]; hence increasing the systemic exposure to statins. We thus also included the *SLCO1B1 c.521 T > C* polymorphism when calculating the statin exposure.

Pharmacogenetic analyses were performed at Diakonhjemmet Hospital, Oslo, Norway using extracted DNA from patient blood samples. Briefly, DNA was extracted from 250 μL blood aliquots and subsequent analysis of the c.521 T > C polymorphism (rs4149056) was carried out using a certified TaqMan-based real-time PCR assay implemented for routine pharmacogenetic analysis at the hospital. The c.521 T > C polymorphism is present on three *SLCO1B1* haplotypes (SLCO1B1*5, *15 and *17), but the phenotypic effect is the same regardless of haplotype [31]. Thus, the patients were divided into three subgroups based on the analysis of the c.521 T > C polymorphism, i.e. homozygous carriers of the c.521C allele, heterozygous carriers, and homozygous carriers of the c.521 T allele (control group; normal *SLCO1B1* genotype).

In pharmacokinetic studies, homozygous carriers of *SLCO1B1 c.521C* have been reported to obtain a systemic exposure of simvastatin, atorvastatin, pravastatin and rosuvastatin of 221, 144, 90 and 87%, respectively, as compared to carriers of the normal (wild type) allele [30]. Heterozygous carriers can be expected to obtain a statin exposure approximately mid-between the two homozygous variants, i.e. the exposure can be expected to increase with a factor of 2.1, 1,7, 1.4 and 1.4 for the four statins, respectively. We adjusted the statin exposure variable accordingly and harmonized the exposure variable to simvastatin equivalents by correcting for the relative potencies of the respective type of statin being used.

### Statistical analysis

We carried out two sets of analyses, one with statin use versus no use as explanatory variable, and the other (limited to the statin users) with calculated statin exposure (as simvastatin equivalents), taking into account statin type, drug-drug interactions and the *SLCO1B1 c.521 T > C* polymorphism. The physical performance and muscle function variables were used as dependent variables. In all analyses with handgrip strength as dependent variable, gender was included as a covariate, whereas gait speed and SPPB were initially analysed with statin exposure as the only explanatory variable. To adjust for factors that can influence physical performance and muscle function as well as prescription, we then included as covariates gender and age as well as the frailty indicators BMI, CIRS (comorbidity), CDR (dementia) and FIM (disability). We decided in advance which independent variables to include and used the ‘enter’ method for multiple regression analysis. We report standardized (β) as well as unstandardized coefficients (B) and their 95% confidence intervals (CI) from the linear regression analyses. B represents the predicted difference in the outcome variable between users and non-users of a statin, or the change in the outcome variable per mg increase in simvastatin equivalent dose.

All the dependent variables were checked to be normally distributed by Q-Q plots before statistical analyses. Degree of multicollinearity was checked by inspection of a correlation matrix between the explanatory variables as well as the variance inflation factors (VIF). We also inspected residual plots and plotted residuals against predicted values in order to assess fit of the regression models. All the analyses were completed using SPSS version 25.

## Results

Descriptive data are reported in Table 1. Numbers of patients fulfilling each of the single EWGSOP criteria for sarcopenia [18] according to grip strength, SPPB score or gait speed are also displayed. Among the statin users (*n* = 87), 54 were treated with simvastatin, 28 with atorvastatin, three with pravastatin and two with rosuvastatin. Among the 54 simvastatin users, 17 were also treated with amlodipine, one with diltiazem and one with amiodarone, thus necessitating exposure adjustment for CYP3A4 inhibition. Genotype was missing for one patient due to lack of sufficient material. Among the 173 genotyped patients, 49 patients (28.2%) were heterozygous for the *SLCO1B1 c.521C* reduced-function variant allele (*TC* genotype), and three (1.7%) were homozygous carriers (CC genotype). This is as expected in a population comprising patients of Caucasian ancestry. The remaining 121 (69.5%) were homozygous for the wild type allele (*TT* genotype). 23 of the heterozygous (**TC**) reduced-function allele carriers, but none of the homozygous (CC) carriers used a statin. When adjusting for CYP3A4 inhibition and *SLCO1B1 c.521 T > C* polymorphism as appropriate, the mean (SD) simvastatin equivalent dose for the 87 statin users was 58.2 (50.7) mg and the median 40.0 mg, with a range from 10 to 336 mg.

**Table 1:** Characteristics of the study population, overall and by use of a statin. Mean (SD) if not otherwise indicated

	Overall *N* = 174	Non-users *N* = 87	Users *N* = 87	Mean difference (95% CI)^a^
Age (years)	83.3 (7.3)	85.7 (7.5)	81.0 (6.4)	−4.7 (−6.8 to − 2.6)
Female, n (%)	118 (67.8)	70 (80.5)	48 (55.2)	*p* < 0.01^b^
BMI (kg/m^2^)	25.2 (5.6)^c^	24.2 (5.4)	26.3 (5.6)	2.1 (0.4 to 3.8)
CIRS (0-56)	16.7 (4.3)	16.3 (4.3)	17.1 (4.2)	0.9 (−0.4 to 2.1)
FIM (18-126)	111.1 (10.8)	110.6 (10.4)	111.7 (11.1)	1.1 (−2.1 to 4.4)
CDR (0-18)	2.3 (3.3)	2.5 (3.3)	2.2 (3.4)	−0.3 (−1.3 to 0.7)
Grip strength (kg)	18.5 (8.1)	15.7 (6.8)	21.4 (8.3)	5.7 (3.4 to 8.0)
Grip strength indicating sarcopenia ^d^, n (%)	105 (60)	61 (70)	44 (51)	*p* = 0.01^b^
SPPB (0-12)	4.6 (3.1)	4.4 (3.0)	4.8 (3.2)	0.4 (−0.6 to 1.3)
SPPB ≤8, n (%)	149 (86)	74 (85)	75 (86)	*p* = 0.8^b^
Gait speed (m/s)	0.62 (0.20)^e^	0.59 (0.21)	0.64 (0.19)	0.06 (−0.01 to 0.11)
Gait speed ≤0.8 m/s, n (%)	128 (79)^e^	68 (83)	60 (75)	*p* = 0.2^b^
SPPB subscore balance (0-4)	1.6 (1.3)	1.6 (1.3)	1.7 (1.4)	0.1 (−0.3 to 0.5)
SPPB subscore chair stand (0-4)	0.8 (1.2)	0.8 (1.2)	0.8 (1.2)	0.0 (−0.3 to 0.4)
n (% of 87) using statin as secondary prevention			64 (74)	

^a^Non-users are reference category

^b^Chi square test

^c^*n* = 168. Six (four statin users) missing due to height not reported

^d^ < 27 kg for men, < 16 kg for women

^e^*n* = 162. Twelve (seven statin users) missing due to amputation or hemiparesis

*CI* Confidence Interval, *BMI* Body mass Index, *CIRS* Cumulative Illness Rating Scale, *FIM* Functional Independence Measure, *CDR* Clinical Dementia Rating Scale (Sum of Boxes), *SPPB* Short Physical Performance Battery

In crude analyses with statin use as explanatory variable, statin users had better scores than non-users on all the dependent variables, and the difference was statistically significant for handgrip strength. Adjusted for gender, the handgrip strength was 2.7 kg higher in statin users than in non-users. When adjusted for all relevant covariates, the estimated difference regarding handgrip strength was reduced to 1.1 kg and was no longer statistically significant. For the other two outcome measures of physical performance, we found no difference (Table 2). As a secondary analysis, we calculated unadjusted and adjusted regression coefficients for the association between statin use and the balance and the chair stand parts of SPPB, separately. Neither for these sub-scores, we found any statistically significant difference between users and non-users. The regression coefficient for balance was 0.13 (95% CI − 0.28 to 0.53) unadjusted and − 0.07 (95% CI − 0.45 to 0.31) when adjusted for all the other covariates (non-users of statins are reference). The regression coefficient for chair stand was 0.02 (95% CI − 0.34 to 0.39) unadjusted and − 0.25 (95% CI − 0.60 to 0.10) when adjusted for the other covariates.

**Table 2:** Linear regression analyses, statin users versus non-users *n* = 174

Dependent variable	Explanatory variables	Unadjusted models^a^			Adjusted model		
		β	B	95% CI for B	β	B	95% CI for B
Grip strength (kg)	Statin use	**0.17**	**2.7**	**1.0 to 4.4**	0.07	1.1	−0.5 to 2.7
	Age (years)	**−0.20**	**−0.22**	**− 0.33 to − 0.11**	**−0.15**	**− 0.17**	**−0.28 to − 0.06**
	Female gender	**−0.74**	**− 12.7**	**−14.5 to − 11.0**	**−0.65**	**− 11.3**	**− 13.0 to − 9.6**
	BMI (kg/m^2^)	**0.23**	**0.33**	**0.19 to 0.48**	**0.16**	**0.24**	**0.10 to 0.37**
	CIRS (0-56)	0.31	0.02	−0.18 to 0.23	0.04	0.09	−0.10 to 0.28
	FIM (18-126)	**0.20**	**0.15**	**0.08 to 0.23**	**0.20**	**0.16**	**0.08 to 0.25**
	CDR (0-18)	**−0.11**	**−0.26**	**−0.51 to − 0.02**	−0.03	− 0.07	−0.32 to 0.18
SPPB (0-12)	Statin use	0.06	0.36	−0.56 to 1.28	−0.02	− 0.14	−0.90 to 0.62
	Age (years)	−0.04	−0.02	− 0.08 to 0.05	−0.08	− 0.03	−0.08 to 0.02
	Female gender	−1.32	−0.87	−1.84 to 0.11	**−0.15**	**− 0.96**	**− 1.7 to − 0.16**
	BMI (kg/m^2^)	**0.17**	**0.09**	**0.01 to 0.17**	0.10	0.05	−0.01 to 0.12
	CIRS (0-56)	**−0.23**	**−1.7**	**−0.27 to − 0.06**	−0.12	− 0.09	−0.18 to 0.00
	FIM (18-126)	**0.58**	**0.17**	**0.13 to 0.20**	**0.68**	**0.21**	**0.17 to 0.25**
	CDR (0-18)	0.04	0.04	−0.10 to 0.18	**0.34**	**0.31**	**0.19 to 0.43**
Gait speed (m/s)	Statin use	0.14	0.06	−0.01 to 0.12	0.05	0.02	−0.04 to 0.08
	Age (years)	**−0.18**	**−0.005**	**− 0.009 to − 0.001**	−0.14	0.00	−0.01 to 0.00
	Female gender	−0.12	−0.05	− 0.12 to 0.01	−0.09	− 0.04	−0.01 to 0.02
	BMI (kg/m^2^)	0.06	0.00	0.00 to 0.01	−0.04	0.00	−0.01 to 0.00
	CIRS (0-56)	**−0.18**	**−0.01**	**− 0.02 to 0.00**	−0.12	− 0.01	−0.01 to 0.00
	FIM (18-126)	**0.50**	**0.01**	**0.008 to 0.013**	**0.57**	**0.01**	**0.01 to 0.02**
	CDR (0-18)	−0.07	0.00	− 0.01 to 0.01	**0.22**	**0.01**	**0.01 to 0.02**

β is the standardized regression coefficient. B is the unstandardized regression coefficient and equals the estimated difference in the dependent variable per unit increase in the explanatory variable

^a^Models for grip strength are adjusted for gender (model with gender as explanatory variable not adjusted). Models for SPPB and gait speed are not adjusted

*CI* Confidence Interval, *BMI* Body mass Index, *CIRS* Cumulative Illness Rating Scale, *FIM* Functional Independence Measure, *CDR* Clinical Dementia Rating Scale (Sum of Boxes), *SPPB* Short Physical Performance Battery

Estimates in bold letters indicate a *p*-value below 0.05

Table 3 presents the distribution of the outcome variables as well as the covariates by quartiles of the statin equivalent variable. When analysing the dose-response relationships within the subpopulation of statin users, we found that all three outcome variables improved with increasing statin exposure. The increase was statistically significant for SPPB and gait speed, both in unadjusted analyses and in analyses adjusted for age, gender and frailty indicators (Table 4). Estimated increase in SPPB was 0.1 point and in gait speed 0.01 m/s per 10 mg increase in simvastatin equivalent exposure. Also for the dose-response relationship, we carried out secondary analyses, calculating unadjusted and adjusted regression coefficients within the subpopulation of statin users for the effect of statin dose upon the balance and the chair stand parts of SPPB, separately. For both, we found a positive association with statin dose that was statistically significant in unadjusted analyses but insignificant when adjusting for all other covariates. The regression coefficient for balance (per mg increase in simvastatin equivalents) was 0.01 (95% CI 0.00 to 0.01) unadjusted and 0.00 (95% CI − 0.01 to 0.01) adjusted, whereas that for chair stand was 0.01 (95% CI 0.00 to 0.01) unadjusted and 0.00 (− 0.01 to 0.01) adjusted.

**Table 3:** Characteristics of the statin users by dosage (in simvastatin equivalents). Mean (SD) if not otherwise indicated *n* = 87

Simvastatin equivalent dose	1st quartile (10 – 30 mg), *n* = 28	2nd quartile (31 – 40 mg), *n* = 22	3rd quartile (41 – 80 mg), *n* = 23	4th quartile (81 – 336 mg), *n* = 14
Age (years)	82.3 (6.4)	81.0 (5.8)	81.0 (6.7)	78.4 (6.6)
Female, n (%)	18 (64)	12 (55)	12 (52)	6 (43)
BMI (kg/m^2^) ^a^	24.7 (4.9)	25.9 (5.7)	26.7 (5.7)	29.4 (5.6)
CIRS (0-56)	16.9 (4.0)	16.8 (4.8)	17.3 (4.3)	18.0 (3.8)
FIM (18-126)	111.4 (12.5)	111.4 (10.4)	114.3 (9.1)	108.6 (12.7)
CDR (0-18)	2.3 (3.6)	1.3 (2.2)	1.9 (2.7)	4.1 (4.9)
Grip strength (kg)	21.0 (9.6)	18.3 (6.9)	22.8 (7.4)	24.9 (7.9)
Grip strength indicating sarcopenia ^b^, n (%)	12 (43)	17 (77)	11 (48)	4 (29)
SPPB (0-12)	4.2 (3.1)	3.9 (2.8)	5.4 (2.9)	6.1 (3.8)
SPPB ≤8, n (%)	24 (86)	21 (96)	21 (91)	9 (64)
Gait speed (m/s) ^c^	0.65 (0.20)	0.58 (0.19)	0.65 (0.16)	0.71 (0.22)
Gait speed ≤0.8 m/s, n (%)	18 (72)	17 (85)	18 (82)	7 (54)

^a^*n* = 83. Four missing due to height not reported

^b^ < 27 kg for men, < 16 kg for women

^c^*n* = 80. Seven missing due to amputation or hemiparesis

*CI* Confidence Interval, *BMI* Body mass Index, *CIRS* Cumulative Illness Rating Scale, *FIM* Functional Independence Measure, *CDR* Clinical Dementia Rating Scale (Sum of Boxes), *SPPB* Short Physical Performance Battery

**Table 4:** Linear regression analyses of statin exposure in simvastatin equivalents, *n* = 87

Dependent variable	Unadjusted models ^a^			Adjusted models ^b^		
	β	B	95% CI for B	β	B	95% CI for B
Grip strength (kg)	0.05	0.01	−0.02 to 0.03	0.03	0.01	−0.02 to 0.03
SPPB (0-12)	**0.26**	**0.02**	**0.00 to 0.03**	**0.19**	**0.01**	**0.00 to 0.02**
Gait speed (m/s)	**0.24**	**0.001**	**0.000 to 0.002**	**0.23**	**0.001**	**0.000 to 0.002**

β is the standardized regression coefficient. B is the unstandardized regression coefficient and equals the estimated change in the dependent variable per milligram increase in statin exposure expressed as simvastatin equivalents, taking into account statin potency, drug-drug interactions and SLCO1B1 genotype. Estimates in bold letters indicate a *p*-value below 0.05

^a^Model for grip strength adjusted for gender

^b^All models adjusted for age, gender, body mass index, Cumulative Illness Rating Scale, Functional Independence Measure and Clinical Dementia Rating Scale

## Discussion

In contrast to our a priori hypothesis, we found no tendency towards impaired physical performance or muscle function in statin users compared to non-users, and neither found we any negative association between statin exposure and the outcome variables among those who used a statin. On the contrary, we found a tendency towards better performance in statin users and in those who were subject to a higher exposure, the latter remaining statistically significant also when adjusting for potential confounders. The positive association between statin use as well as increasing statin exposure and the outcome measures may suggest that use of statins actually improves the physical function in older patients subjected to complex drug treatment.

However, it is important to be aware the naturalistic setting of our study, and the results should be interpreted with caution. A possible explanation for our surprising findings might be that there exists residual confounding that was not measured and thus not adjusted for. Clinicians may interpret patients with impaired physical performance as frail and therefore avoid statin prescriptions or prescribe a lower dose. We were, however, able to adjust for the essential frailty indicators underweight, multimorbidity, cognitive failure and disability, thus improving the validity of our results. As expected, this adjustment deflated the association between statin use and the dependent variables, but the dose-response relationship among the statin users remained at approximately the same magnitude and was still statistically significant after adjustment. According to the protocol for the intervention trial [14], patients were ineligible if they had an expected remaining lifetime of less than 6 months or were expected to move permanently to a nursing home during the same period. Thus, the frailest patients, that might be those most prone to negative effects of statins, were not included.

A potential causal explanation of the present findings might be that statin use per se exhibits positive effects on the muscular system [1], possibly slowing down a natural loss of physical function. Evidence is emerging that statins have anti-inflammatory properties, reducing pro-inflammatory cytokine levels [32, 33]. A recent secondary analysis of a strength training trial in older adults reported better effect of the training upon fatigue resistance among statin users than among non-users [34]. When comparing users and non-users of statins, we found a statistically significant difference only for grip strength and only in the unadjusted model. For the dose-response relationship within the group of statin users, on the other side, the statistical significance remained also in adjusted models, and was most obvious regarding SPPB score and gait speed. The lack of statistical significance in some of the tests might be due to limited statistical power, as the tendency was in the same direction for all comparisons including the sub-scores of SPPB. Our study is, by all means, hypothesis-generating. Possible positive or negative effects of statins upon muscle function should be investigated further in prospective studies measuring physical performance after vs. before the initiation or withdrawal of different doses of statins.

Our study has certain limitations. It is cross-sectional, and thus not feasible to establish causality between statin use and physical performance. The study was powered for the estimated effect size in the main trial [14] and not for the present topic. Accordingly, the number of participants is low. Moreover, participants were not asked about adherence to their prescribed statin therapy nor about subjective adverse effects, drug concentrations were not measured, and we do not have detailed data on the patients’ diagnoses. These are obvious limitations. A previous study reported that one third of statin users were nonadherent to the treatment [35]. Non-compliance is likely to be non-random, as patients who experience subjective side effects are more likely to be non-compliant. Such effects might mask a possible association between statin use and impaired physical performance.

The study has, however, also certain strengths, most importantly our ability to adjust for relevant aspects of frailty like dementia, disability, and underweight, which might else have confounded possible associations. We also adjusted for known pharmacokinetic interactions as well as for pharmacogenetic variation. Moreover, the fact that all our participants had their medication administered by the home nursing service indicates a higher adherence than else observed [36]**.**

## Conclusion

Statin use did not seem to affect grip strength, gait speed nor SPPB scores negatively in home-dwelling patients aged 70+ who used at least seven medications and had their medication administered by the home nursing service. Our study suggests the opposite, a possible positive effect of statin use and exposure on physical performance and muscle function that should be further studied.

## Data Availability

The datasets presented in this article are not readily available because of Norwegian regulations and conditions for informed consent. Requests to access the dataset should be directed to TBW (t.b.wyller@medisin.uio.no).

## Abbreviations

BMI: Body Mass Inex
CDR: Clinical Dementia Rating Scale
CI: Confidence Interval
CIRS: Cumulative Illness Rating Scale
COOP: Cooperation between geriatricians and general practitioners for improved pharmacotherapy in home-dwelling elderly people receiving polypharmacy study
CYP: Cytocrom P
FIM: Functional Independence Measure
SD: Standard Deviation
SPPB: Short Physcal Performance Battery
VIF: Variance Inflation Factor
